# Relative efficacy and safety of mesenchymal stem cells for osteoarthritis: a systematic review and meta-analysis of randomized controlled trials

**DOI:** 10.3389/fendo.2024.1366297

**Published:** 2024-06-10

**Authors:** Xiaoyuan Tian, Zhenan Qu, Ying Cao, Bocheng Zhang

**Affiliations:** ^1^ Second Affiliated Hospital, Dalian Medical University, Dalian, Liaoning, China; ^2^ Department of Orthopaedics, Affiliated Zhongshan Hospital of Dalian University, Dalian, Liaoning, China; ^3^ Department of Orthopaedics, Second Affiliated Hospital, Dalian Medical University, Dalian, Liaoning, China

**Keywords:** mesenchymal stem cell, osteoarthritis, randomized controlled trials, meta-analysis, systematic review

## Abstract

**Introduction:**

The aim of this meta-analysis was to evaluate the efficacy and safety of mesenchymal stem cells (MSCs) for the treatment of knee osteoarthritis (OA).

**Methods:**

The PubMed, Embase, Cochrane Central Register of Controlled Trials, Scopus and Web of Science databases were searched from inception to May 6, 2024 to identify randomized controlled trials that compared MSCs and placebo or other nonsurgical approaches for treating OA. Two investigators independently searched the literature and extracted data, and conventional meta-analyses were conducted with Review Manager 5.3. The outcomes included pain relief, functional improvement, and risk of adverse events (AEs).

**Results:**

A total of 18 articles were included. Overall, MSCs were superior to placebo in terms of relieving pain and improving function at the 12-month follow-up. However, the differences in treatment-related AEs were not significant.

**Conclusion:**

MSCs may relieving pain and improving function of OA. The limitations of this study include the high heterogeneity of the included studies. Additionally, the follow-up time in the included studies was relatively short, so more clinical trials are needed to predict the long-term efficacy and safety of MSCs.

**Systematic review registration:**

https://doi.org/10.17605/OSF.IO/5BT6E, identifier CRD42022354824.

## 1 Introduction

Osteoarthritis (OA) is the most common bone and joint disease among elderly individuals; it affects approximately 500 million people worldwide and causes joint pain, swelling, stiffness and joint deformity. This highly debilitating condition represents a major global public health concern (1). The pathogenesis of OA is intricate and heterogeneous; OA is characterized by cartilage degradation and alterations in cartilage composition that impact its mechanical properties, concomitant with the deformation of articular cartilage, leading to increased stiffness and loss of elastic behavior (2). Moreover, it is characterized not only by cartilage loss but also by fibrosis, synovial hyperplasia, subchondral bone remodelling, and meniscal degeneration (3). Moreover, the infrapatellar fat pad exhibits increased fibrosis, hypervascularization and augmented lymphocyte infiltration (4). Nonsteroidal anti-inflammatory drugs (NSAIDs) are currently the main treatment option for OA patients with persistent pain or moderate or severe pain (5), but these drugs are associated with several adverse events (AEs), such as gastrointestinal bleeding and cardiovascular complications. Furthermore, NSAIDs rarely achieve satisfactory therapeutic effects for patients with advanced OA. Moreover, there are no approved pharmacological interventions, biological therapies or procedures for preventing the pathological progression of OA. Total joint replacement (TJR) can successfully relieve pain and improve function, but it is accompanied by substantial risks such as thrombosis and infection (6). Furthermore, TJR can lead to costly hospital care, physical therapy, and rehabilitation; therefore, it is always a last resort for OA treatment (7, 8).

In the past ten years, cell therapy, especially therapy with mesenchymal stem cells (MSCs), has gradually attracted increasing amounts of attention. MSCs are pluripotent stem cells that can differentiate into multiple lineages, including mesenchymal and nonmesenchymal lineages. MSCs are mainly derived from bone marrow (BM) (9), adipose tissue (AD) (10) and umbilical cord (UC) blood (11). Numerous preclinical studies have demonstrated the anti-inflammatory and antiapoptotic effects of these compounds (12). Additionally, MSCs improve cartilage regeneration in OA (13). In recent years, some clinical studies have assessed MSCs in the treatment of OA. These studies have shown that MSCs relieve pain, improve function and promote cartilage repair (14, 15). Nevertheless, a variety of contradictory clinical outcomes have been reported in the literature. For example, one study revealed that after intra-articular injection of 4 different concentrations of allogeneic BM-MSCs, no significant improvements in the knee joint function score or imaging results were noted compared with those of a placebo (16).

Some recent systematic reviews or meta-analyses obtained similar results (17, 18), but some meta-analyses have suggested that MSCs do not have any advantage compared with placebo (19–21). Additionally, research on MSCs has come under heavy criticism; several MSC-based clinical trials have failed on primary end points, causing many to question whether these stem cells should continue to be studied (22). Nonetheless, many new studies evaluating the therapeutic effect of MSCs were reported in 2023 (23–25). Therefore, this review summarizes and updates the results of studies on the use of MSCs for treating OA. Additionally, a meta-analysis was performed to further evaluate the efficacy of MSCs for treating OA.

## 2 Methods

### 2.1 Protocol and registration

This systematic review and meta-analysis study was conducted in accordance with the Preferred Reporting Items for Systematic Reviews and Meta-analysis (PRISMA) guidelines (26), and the protocol was registered on Open Science Framework (https://doi.org/10.17605/OSF.IO/5BT6E).

### 2.2 Eligibility criteria

The inclusion criteria were as follows: randomized controlled trials (RCTs) assessing the administration of MSCs for treating OA based on the American College of Rheumatology criteria (27) and a Kellgren-Lawrence grade of at least 1 (28); the intervention group received MSCs as monotherapy, with no restrictions based on dosage, route of administration or time of MSC application; the control group received a blank treatment or isopycnic placebo; the outcome indicators were the visual analogue scale (VAS) (29) or the total Western Ontario and McMaster Universities Osteoarthritis Index (WOMAC) (30); safety was assessed as adverse events (AEs); and studies published in English.

The following studies were excluded: secondary analyses, including pooled analyses; reviews or conference abstracts; studies of MSCs combined with other surgeries, such as arthroscopic debridement and high tibial osteotomy (HTO); studies with a follow-up duration shorter than 6 months; and abstracts (insufficient data).

### 2.3 Information sources

We systematically searched the Cochrane Central Register of Controlled Trials, PubMed, Embase, Scopus and Web of Science databases from inception to May 6, 2024.

### 2.4 Search

We used a combination of relevant terms, including “mesenchymal stem cells”, “osteoarthrosis”, “placebo”, and “randomized controlled trial” (**Supplementary Table 1**). In addition, the reference lists of both the included studies and relevant reviews were manually searched to identify additional eligible studies. We included only articles published in English.

### 2.5 Study selection

The study selection process was performed independently by two review authors (Z.A. Qu and X.Y. Tian). We obtained the full texts of the studies to determine their eligibility. If there were multiple reports that described the same trial, only the most recent or complete study was included.

### 2.6 Data collection process

Relevant data from the selected studies were independently extracted in accordance with the inclusion criteria by two authors (X.Y. Tian and Z.A. Qu). A third author (B.C. Zhang) was consulted to resolve any disagreements regarding study selection and data extraction. If the means and standard deviations were not reported in the text of the articles, we extracted the values from the diagrams and tables as needed.

### 2.7 Data items

The following data were extracted from the included studies: author’s name, year of publication, patient information (including sex, mean age), MSC type, regimen in the intervention and control groups, and follow-up duration.

The primary outcome measures of interest were the mean change in the WOMAC score and the VAS score between baseline and the endpoint. The secondary outcome measures were the incidence rates of treatment-related AEs, such as arthralgia and swelling.

### 2.8 Quality assessment

Two review authors (X.Y. Tian and Z.A. Qu) independently assessed the risk of bias for each study using the Cochrane risk of bias assessment tool (31). The tool assesses seven specific domains: sequence generation, allocation concealment, blinding of participants and personnel, blinding of outcome assessment, incomplete outcome data, selective outcome reporting and ‘other sources of bias’. Each domain was scored as having a low risk of bias, high risk of bias or unclear risk of bias. Disagreements were resolved by discussion and consensus.

### 2.9 Statistical analysis

A conventional meta-analysis was conducted to compare MSCs with placebo using Review Manager 5.3 (Cochrane Collaboration, Oxford, UK). Dichotomous data were examined using risk ratios (RRs) and 95% confidence intervals (CIs) based on the number of events in the control and intervention groups of each study. Continuous data were analyzed using the mean differences (MDs) and 95% CIs between the MSC and control groups. The WOMAC and VAS scores were converted to a common scale from 0 (no pain or disability) to 100 (worst possible pain or disability) before meta-analysis. The heterogeneity of the effect size across the studies was tested using the chi-square test (p < 0.1 was considered heterogeneous) and I^2^ statistic (I^2^ > 50% was considered heterogeneous). If significant heterogeneity existed between studies, a random effects model was used; otherwise, a fixed effects model was used. Subgroup analysis was performed based on the dose and type of MSCs, mean MSC counts per injection and autologous or allogenic MSCs. A sensitivity analysis was performed to examine the reliability of the results. When a control group served multiple experimental groups, the number of participants in the control group was divided by the number of experimental groups. The overall effect was tested using a Z score with the significance set at p < 0.05. We used funnel plots to assess publication bias if more than 10 included trials examined a particular outcome.

## 3 Results

### 3.1 Study selection

**Figure 1:**
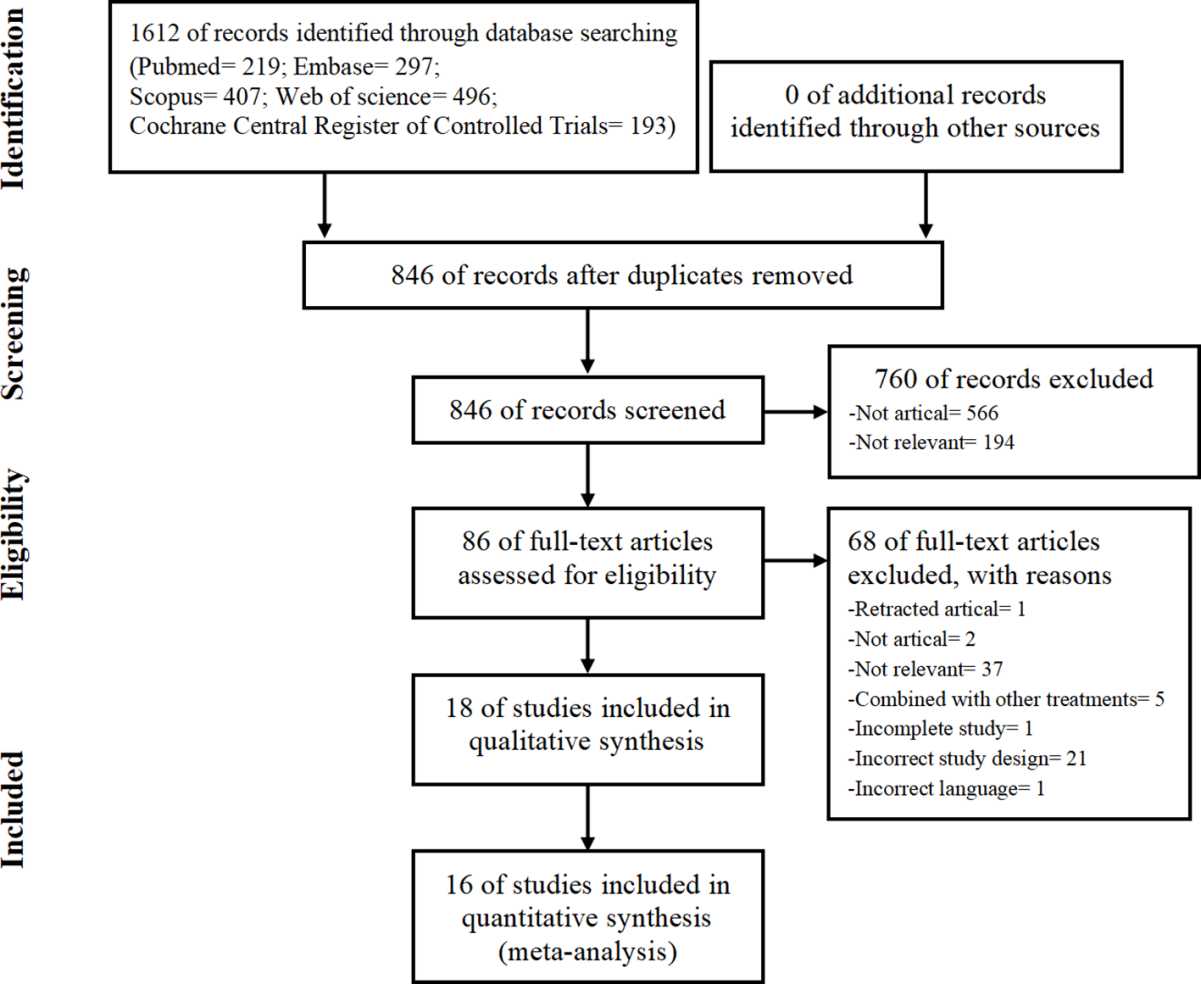
Preferred Reporting Items for Systematic Reviews and Meta-Analysis (PRISMA) flowchart showing the procedure used to search for and identify the included studies.

The PRISMA flow chart of study selection is shown in **Figure 1**. We initially identified 1,612 studies from database searches. After removing duplicates, 846 studies remained. We then excluded 760 studies based on the titles and abstracts. We assessed the full texts of the remaining 86 papers and excluded 68 studies (details in **Supplementary Table 2**). Ultimately, we included 18 studies (14–16, 23–25, 32–43) in the systematic review and 16 studies (14–16, 23–25, 32–34, 36, 37, 39–43) in the meta-analysis.

### 3.2 Study characteristics

The characteristics of the included studies are shown in **Table 1**. This systematic review included 18 eligible studies with a total sample size of 1,174 participants. A total of 633 patients were included in the intervention group. Among the cell sources, 3 studies examined allogeneic AD-MSCs (14, 25, 39), 4 studies examined autologous AD-MSCs (24, 32, 34, 41), 3 studies examined allogeneic BM-MSCs (16, 23, 40), 5 studies examined autologous BM-MSCs (15, 33, 35–37), and 3 studies examined allogeneic UC-MSCs (38, 42, 43). In the included studies, the average number of MSCs per injection ranged from 1× 10^6^ to 150 × 10^6^. Fourteen studies compared the effects of a single dose of MSCs, with seven studies utilizing a dosage range of 50–100 × 10^6^ and an additional seven studies employing a dosage less than 50 × 10^6^. The remaining four studies examined varying doses of MSCs.

**Table 1:** Characteristics of the included comparisons.

Study (year)	Cell source	Group (Dose)	N	Mean age (year)	Female (%)	K-L grade	Study duration	Trial num.	Outcome index
Vega (2015)	Allogeneic	Single injection (40 × 10^6^ BM-MSCs)	15	56.6	9 (60.0)	II-IV	12 months	NCT01586312	1. VAS 2. WOMAC 3. MRI
		Placebo (Hyaluronic acid)	15	57.3	10 (66.7)				4. SF-12 5. Lequesne index 6. AEs
Gupta (2016)	Allogeneic	Single injection (25 × 10^6^ BM-MSCs)	10	58.1	7 (70.0)	II-III	12 months	NCT01453738	1. VAS 2. WOMAC
		Single injection (50 × 10^6^ BM-MSCs)	10	57.3	8 (80.0)				3. WORMS 4. ICOAP 5. AEs
		Single injection (75 × 10^6^ BM-MSCs)	10	55	8 (80.0)				
		Single injection (150 × 10^6^ BM-MSCs)	10	54	5 (50.0)				
		Placebo (5%serum albumin+10%DMSO)	20	57.0	17 (85.0)				
Espinosa (2016)	Autologous	Single injection (10 × 10^6^ BM-MSCs)	10	65.9	6 (60.0)	II-IV	12 months	NCT02123368	1. VAS 2. WOMAC
		Single injection (100 × 10^6^ BM-MSCs)	10	57.8	2 (20.0)				3. WORMS 4. AEs
		Placebo (Hyaluronic acid)	10	60.3	3 (30.0)				
Emadedin (2018)	Autologous	Single injection (40 × 10^6^ BM-MSCs)	19	51.7	7 (36.8)	II-IV	6 months	NCT01504464	1. VAS 2. WOMAC
		Placebo (saline)	24	54.7	9 (37.5)				3. Physical examination 4. AEs
Soltani (2018)	Allogeneic	Single injection (50 × 10^6^ placenta-MSCs)	10	57.5	9 (90.0)	II-IV	6 months	IRCT2015101823298N	1. VAS 2. KOOS 3. MRA
		Placebo (saline)	10	55.8	9 (90.0)				4. Physical examination 5. AEs
Matas (2018)	Allogeneic	Single injection (20 × 10^6^ UC-MSCs)	10	56.1	6 (60.0)	II-III	12 months	NCT02580695	1. WOMAC 2. VAS
		Two injections (20 × 10^6^ UC-MSCs)	10	56.7	5 (50.0)				3. SF-36 4. WORMS 5. AEs
		Placebo (Hyaluronic acid)	9	54.8	5 (55.0)				
Kuah (2018)	Allogeneic	Single injection (3.9 × 10^6^ AD-MSCs)	8	50.8	2 (25)	I-III	12 months	ACTRN12615000439549	1.VAS 2. WOMAC
		Single injection (6.7 × 10^6^ AD-MSCs)	8	55.0	3 (37.5)				3. AQoL-4D 4. MOAKS 5. AEs
		Placebo (culture media and cryopreservative)	4	55.0	3 (75)				
Freitag (2019)	Autologous	Single injection (100 × 10^6^ AD-MSCs)	10	54.6	3 (30.0)	II-III	12 months	ACTRN12614000814673	1. NPRS 2. KOOS
		Swo injections (100 × 10^6^ AD-MSCs)	10	54.7	6 (60.0)				3. WOMAC 4. MOAKS 5. AEs
		Placebo (conservative management)	10	51.5	5 (50.0)				
Lee (2019)	Autologous	Single injection (100 × 10^6^ AD-MSCs)	12	62.2	9 (75.0)	II-IV	6 months	NCT 02658344	1. WOMAC 2. VAS 3. KOOS
		Placebo (saline)	12	63.2	9 (75.0)				4. Physical examination 5. AEs
Lu (2019)	Autologous	Two injections (50 × 10^6^ AD-MSCs)	26	55.0	23 (88.5)	I-III	12 months	NCT02162693	1. WOMAC 2. VAS
		Placebo (Hyaluronic acid)	26	59.6	23 (88.5)				3. SF-36 4. MRI 5. AEs
Bastos (2019)	Autologous	Single injection (40 × 10^6^ BM-MSCs)	16	55.7	6 (37.5)	I-IV	12 months	Not described	1. KOOS 2. Knee range of motion
		Placebo (Corticosteroid)	17	55.9	8 (47.1)				3. Synovial fluid cytokine analysis
Chen (2021)	Allogeneic	Single injection (16 × 10^6^ AD-MSCs)	17	67.7	14 (82.4)	II-III	24 months	NCT02784964	1. WOMAC 2. VAS
		Single injection (32 × 10^6^ AD-MSCs)	17	68.6	15 (88.2)				3. KSCRS 4. AEs
		Single injection (64 × 10^6^ AD-MSCs)	15	64.9	12 (80.0)				
		Placebo (Hyaluronic acid)	8	70.5	5 (62.5)				
Espinosa (2020)	Autologous	Single injection (100 × 10^6^ BM-MSCs)	24	56.0	7 (29.2)	II-IV	12 months	NCT02365142	1. VAS 2. WOMAC
		Placebo (Platelet Rich Plasma)	26	54.6	10 (38.5)				3. WORMS 4. AEs
Ho (2022)	Autologous	Single injection (1 × 10^6^ BM-MSCs)	10	56.7	4 (40.0)	II-III	12 months	CUHK_CCT00469	1. VAS 2. KSS 3. KSFS 4. WOMAC
		Placebo (Hyaluronic acid)	10	59.1	8 (80.0)				5. SF-36 questionnaire 6. MRI 7. AEs
Sadri (2023)	Allogeneic	Single injection (100 × 10^6^ AD-MSCs)	20	52.85	18 (90.0)	II-III	12 months	IRCT20080728001031N23	1. WOMAC 2. VAS 3. KOOS
		Placebo (saline)	20	56.1	18 (90.0)				4. SF-36 5. MRI 6. AEs
Kim (2023)	Autologous	Single injection (100 × 10^6^ AD-MSCs)	125	63.7	86 (68.8)	III	6 months	NCT03990805	1. WOMAC 2. VAS 3. KOOS
		Placebo (saline)	127	63.8	101 (79.5)				4. SF-36 5. WORMS 6. AEs
Gupta (2023)	Allogeneic	Single injection (25 × 10^6^ BM-MSCs)	73	51.6	47 (64.4)	II-III	12 months	No. CTRI/2018/09/015785	1. WOMAC 2. VAS
		Placebo (5%serum albumin+10%DMSO)	73	53.6	51 (69.9)				3. MRI 4. AEs
Mautner (2023)	Allogeneic	Single injection (20 × 10^6^ UC-MSCs)	118	57.9	65 (55)	II-IV	12 months	NCT03818737	1. VAS 2. KOOS
		Placebo (Corticosteroid)	120	58.3	71 (59)				3. MRI 4. AEs

AD, Adipose tissue; BM, Bone marrow; UC, Umbilical cord blood; MSC, Mesenchymal stem cell; NRPS, Numeric Pain Rating Scale; KOOS, Knee Injury and Osteoarthritis Outcome Score; WOMAC, Western Ontario and McMaster Universities Arthritis; MOAKS, MRI Osteoarthritis Knee Score; VAS, Visual Analogue Scale; ICOAP, Intermittent and Constant Osteoarthritis Pain; WORMS, Whole-organ Magnetic Resonance Imaging Score; KSS, Knee Society Score; KSFS, Knee Society Function Score; MRI, Magnetic Resonance Image; KSCRS, Knee Society Clinical Rating System; AE, Adverse Events.

One study had a follow-up duration of 24 months (14), 13 studies had a follow-up duration of 12 months (15, 16, 23, 25, 32, 35–37, 39–43), and 4 studies had a follow-up duration of 6 months (24, 33, 34, 38). Seventeen studies were registered, and one study was not registered (35). Most of the included participants had Kellgren-Lawrence grades of 2 (28.8%) and 3 (55.6%).

### 3.3 Quality assessment

**Figure 2:**
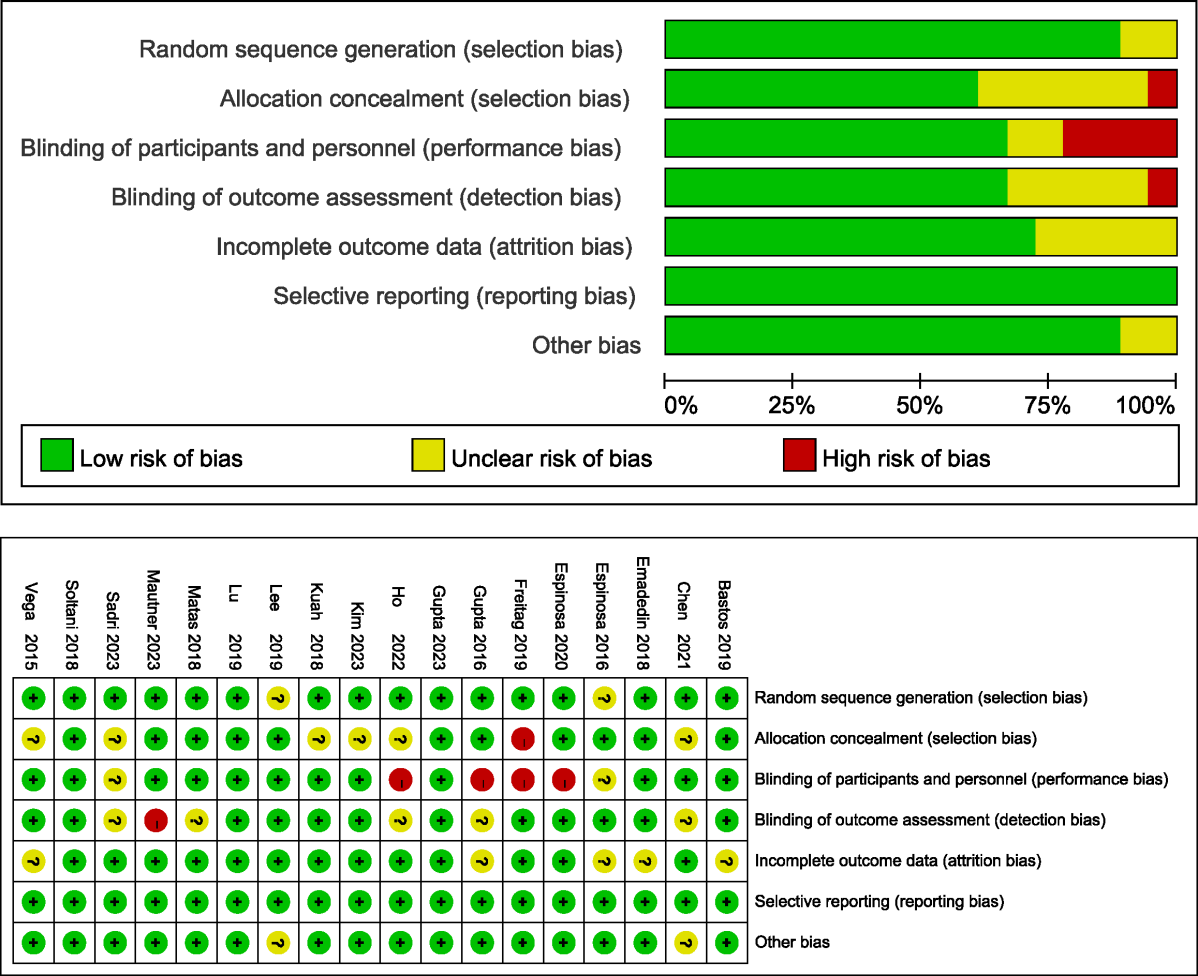
Risk of bias in the selected studies.

The risk of bias assessment for all of the included studies is shown in **Figure 2**. For 3 studies, all domains were judged as having a low risk of bias (23, 38, 41). Randomized sequence generation was not implemented adequately in 2 studies (34, 36); however, all of the included studies were RCTs. Allocation concealment was not implemented adequately in 7 studies (14, 15, 24, 25, 32, 39, 40). Twelve studies successfully reported blinding of participants (14, 23, 24, 33–35, 38–43), and the study personnel were at low risk of performance bias. Twelve studies reported the blinding of outcome assessors, thus leading to a low risk of detection bias (23, 24, 32–41). Five studies did not present loss to follow-up data and thus had an unclear risk of attrition bias (16, 33, 35, 37, 40). All studies exhibited a low risk of reporting bias.

### 3.4 WOMAC index

**Figure 3:**
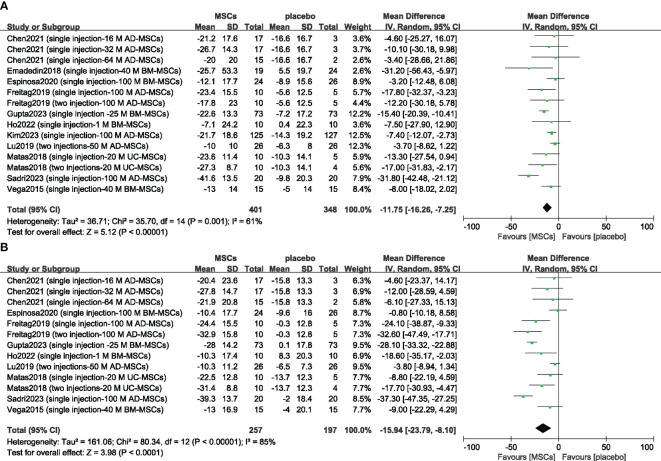
Forest plots of the mean change in the Western Ontario and McMaster Universities Osteoarthritis Index at 6 months **(A)** and 12 months **(B)**. CI indicates confidence interval.

Eleven included studies evaluating efficacy used a reduction in the WOMAC score as the primary or secondary outcome (14, 15, 23–25, 32, 33, 37, 40–42). First, we compared the mean change in the 6-month WOMAC between the MSC group and the placebo group. The reduction in the MSC group was significantly greater than that in the placebo group (n = 749; MD = -11.75; 95% CI = -16.26–7.25; p < 0.00001; I^2^ = 61%) (**Figure 3A**). Similar results were obtained for the 12-month WOMAC (n = 454; MD = -15.94; 95% CI = -23.79–8.10; p < 0.00001; I^2^ = 85%) (**Figure 3B**). Sensitivity analysis was conducted to examine the robustness of these results, and the efficacy was not substantially altered after removing any one study. The visual cues in the funnel plots indicated no conclusive evidence of publication bias (**Supplementary Figure 1**).

### 3.5 VAS score

**Figure 4:**
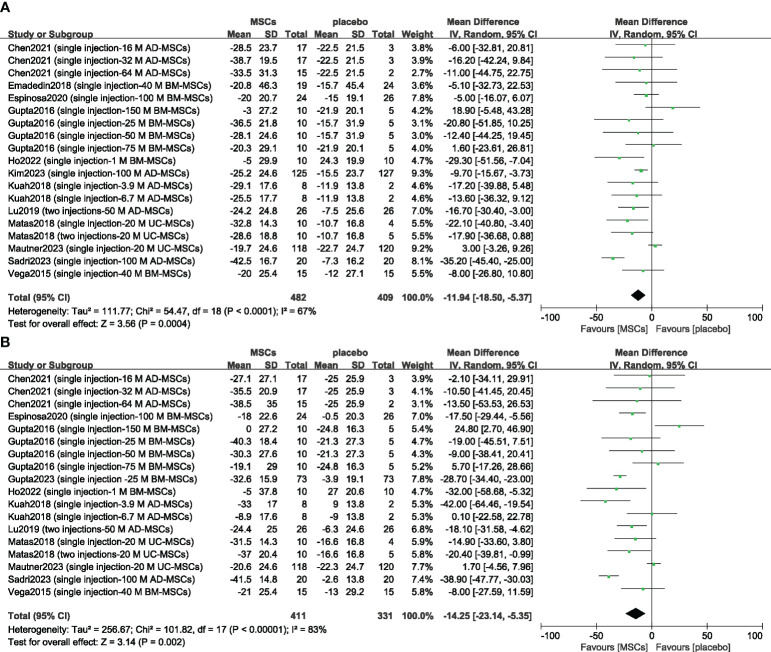
Forest plots of the mean change in the Visual Analogue Scale at 6 months **(A)** and at 12 months **(B)**. CI indicates confidence interval.

Thirteen studies evaluated the analgesic effect of MSCs using the VAS score (14–16, 23–25, 33, 37, 39–43). The improvement in the VAS score significantly differed between the MSC group and the placebo group at 6 months (n = 891; MD = -11.94; 95% CI = -18.50–5.37; p = 0.0004; I^2^ = 67%) (**Figure 4A**). Similar results were obtained for the 12-month VAS score (n = 742; MD = -14.25; 95% CI = -23.14–5.35; p = 0.002; I^2^ = 83%) (**Figure 4B**). Sensitivity analysis was conducted to examine the robustness of these results, and the efficacy was not substantially altered after removing any one study. The visual cues in the funnel plots indicated no conclusive evidence of publication bias (**Supplementary Figure 2**).

### 3.6 Safety

**Figure 5:**
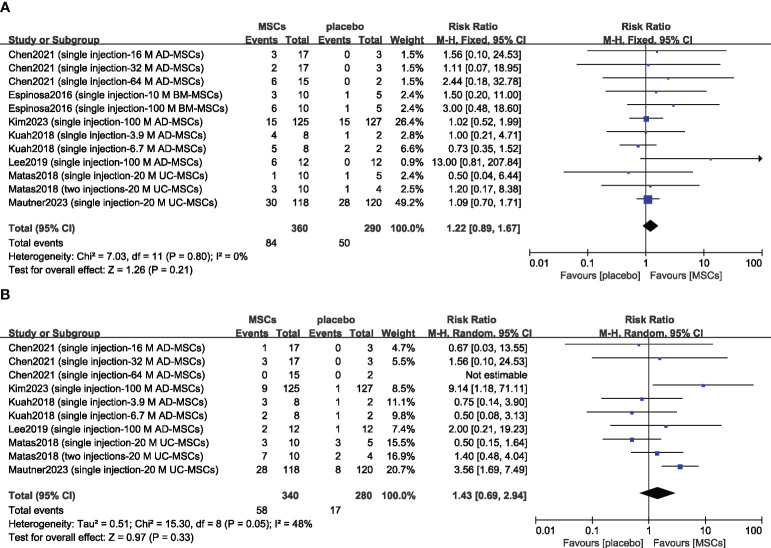
Forest plots of patient treatment-related adverse events: arthralgia **(A)** and swelling **(B)**. CI indicates confidence interval.

Previous studies have suggested that transient arthralgia and swelling can occur after MSC injection, so we evaluated the safety of MSCs by examining the incidence rates of treatment-related arthralgia and swelling. The risks of arthralgia (n = 650; RR = 1.22; 95% CI = 0.89–1.67; p = 0.21; I^2^ = 0%) (**Figure 5A**) and joint swelling (n = 620; RR = 1.43; 95% CI = 0.69–2.94; p = 0.33; I^2^ = 48%) (**Figure 5B**) were greater in the MSC group, but there was no significant difference compared with the placebo group. Sensitivity analysis was conducted to examine the robustness of these results, and the findings were not substantially altered after removing any one study. The visual cues in the funnel plots indicated no conclusive evidence of publication bias (**Supplementary Figure 3**).

### 3.7 Subgroup analysis

Based on the results described above, we analyzed the WOMAC index and the VAS score across different subgroups stratified according to the following variables: MSC type (BM, AD or UC), MSC source (autologous or allogeneic), and mean MSC count per injection (low: 0–49×10^6^ cells or high: greater than 50×10^6^ cells) (**Tables 2**, **3**).

**Table 2:** Subgroup analysis of the Western Ontario and McMaster Universities Osteoarthritis Index.

Analysis	No. of trials	No. analyzed	MD (95% CI)	p	I^2^
6-Month WOMAC	15	749	-11.75 [-16.25, -7.25]	<0.00001	61%
Subgroup analysis					
MSC cell type					
BM-MSC	5	289	-10.95 [-18.00, -3.90]	0.002	52%
AD-MSC	8	431	-11.79 [-19.03, -4.55]	0.001	71%
UC-MSC	2	29	-15.07 [-25.34, -4.80]	0.004	0%
MSC cell count					
Low	9	360	-10.80 [-16.03, -5.57]	<0.0001	47%
High	6	389	-13.05 [-22.42, -3.68]	0.006	76%
MSC cell source					
Autologous	7	447	-7.20 [-11.39, -3.00]	0.0008	27%
Allogeneic	8	302	-14.94 [-21.17, -8.71]	<0.00001	48%
12-Month WOMAC	13	454	-15.94 [-23.79, -8.10]	<0.0001	85%
Subgroup analysis					
MSC cell type					
BM-MSC	4	246	-14.31 [-29.66, 1.04]	0.07	89%
AD-MSC	7	179	-17.64 [-30.52, -4.75]	0.007	87%
UC-MSC	2	29	-13.30 [-22.71, -3.89]	0.006	0%
MSC cell count					
Low	8	317	-13.16 [-22.50, -3.82]	0.006	85%
High	5	137	-20.49 [-37.04, -3.94]	0.02	88%
MSC cell source					
Autologous	5	152	-14.48 [-25.77, -3.19]	0.01	81%
Allogeneic	8	302	-17.15 [-25.93, -8.37]	0.0001	75%

MD, mean difference; CI, confidence interval; WOMAC, Western Ontario and McMaster Universities Osteoarthritis Index; MSC, mesenchymal stem cell; AD-MSC, adipose tissue-derived mesenchymal stem cell; BM-MSC, bone marrow-derived mesenchymal stem cell; UC-MSC, umbilical cord blood-derived mesenchymal stem cell.

**Table 3:** Subgroup analysis of the visual analogue scale.

Analysis	No. of trials	No. analyzed	MD (95% CI)	p	I^2^
6-Month VAS	19	891	-11.94 [-18.50, -5.37]	0.0004	67%
Subgroup analysis					
MSC cell type					
BM-MSC	8	203	-6.90 [-16.00, 2.20]	0.14	27%
AD-MSC	8	421	-17.15 [-26.51, -7.79]	0.0003	62%
UC-MSC	3	267	-10.59 [-28.78, 7.60]	0.25	79%
MSC cell count					
Low	13	502	-12.58 [-19.84, -5.32]	0.0007	45%
High	6	389	-8.87 [-22.47, 4.74]	0.20	83%
MSC cell source					
Autologous	5	417	-10.78 [-16.52, -5.04]	0.0002	15%
Allogeneic	14	474	-11.55 [-21.39, -1.71]	0.02	74%
12-Month VAS	18	742	-14.25 [-23.14, -5.35]	0.002	83%
Subgroup analysis					
MSC cell type					
BM-MSC	8	306	-11.36 [-24.30, 1.58]	0.09	78%
AD-MSC	7	169	-20.59 [-34.51, -6.68]	0.004	69%
UC-MSC	3	267	-8.97 [-24.25, 6.32]	0.25	70%
MSC cell count					
Low	13	605	-15.78 [-25.58, -5.97]	0.002	80%
High	5	137	-8.99 [-32.06, 14.07]	0.44	89%
MSC cell source					
Autologous	3	122	-19.20 [-27.68, -10.72]	<0.00001	0%
Allogeneic	15	620	-12.46 [-23.15, -1.77]	0.02	86%

MD, mean difference; CI, confidence interval; VAS, Visual Analogue Scale; MSC, mesenchymal stem cell; AD-MSC, adipose tissue-derived mesenchymal stem cell; BM-MSC, bone marrow-derived mesenchymal stem cell; UC-MSC, umbilical cord blood-derived mesenchymal stem cell.

In the subgroup analysis of the WOMAC, 11 studies were synthesized at the 6-month follow-up, and 9 studies were synthesized at the 12-month follow-up. No significant difference in the 6-month WOMAC score was observed in the subgroup analyses when the data were stratified by MSC type and mean MSC count per injection. Overall, allogeneic MSCs may have a better curative effect than autologous MSCs (MD = -14.94, 95% CI = -21.17–8.71 vs. MD = -7.20, 95% CI = -11.39–3.00; p = 0.04, I^2^ = 75.5%). Subgroup analyses did not reveal any significant differences in the 12-month WOMAC index score between subgroups. Additionally, among the BM-MSC group, the 12-month WOMAC index score was not significantly different between the MSC group and the control group (p = 0.07).

According to the subgroup analysis of the VAS score, 12 studies reported a 6-month follow-up, and 11 studies reported a 12-month follow-up. No significant difference in the 6-month VAS score was observed in the subgroup analyses when the data were stratified by MSC type, MSC source or mean MSC count per injection. Additionally, among the UC-MSC group, BM-MSC group and high cell count group, the 6-month VAS score did not significantly differ between the MSC group and the control group (UC-MSC group: p = 0.25; BM-MSC group: p = 0.14; high cell count group: p = 0.20). Similar results were obtained for the 12-month VAS score. No significant difference in the 12-month VAS score was observed in the subgroup analyses. The 12-month VAS score of the UC-MSC group, BM-MSC group and high cell count group did not differ from that of the placebo group (UC-MSC group: p = 0.25; BM-MSC group: p = 0.09; high cell count group: p = 0.44).

## 4 Discussion

In this systematic review and meta-analysis, the safety and effectiveness of MSCs for the treatment of OA were evaluated. A total of 18 RCTs involving 1,174 participants were included. Compared with the placebo, the use of MSCs significantly improved the WOMAC score and VAS score at the 6-month follow-up and 12-month follow-up. Additionally, compared with those in the placebo group, there was no increase in the risk of treatment-related arthralgia or swelling. According to the subgroup analysis, the UC-MSC and BM-MSC groups showed no significant differences from the placebo group in terms of functional improvement or analgesia. Regarding cell source, allogeneic MSCs may exert more beneficial effects on OA. A high cell number may not increase the analgesic effect of the treatment compared to a low cell number.

Previous reviews on the efficacy of MSCs in the treatment of OA found that MSCs can significantly improve function, reduce pain, and improve quality of life compared with placebo (44, 45). A meta-analysis published in 2022 included 28 RCTs involving 1494 participants and suggested that MSCs can significantly improve WOMAC pain, WOMAC stiffness, WOMAC physical function and VAS scores for at least 12 months (46). However, the results from a previous meta-analysis showed that MSC has no obvious advantage compared with placebo (20). In 2021, a network meta-analysis of 43 studies including 5554 patients compared the efficacy of hyaluronic acid (HA), steroids, platelet-rich plasma (PRP) and AD-MSCs in the treatment of OA. For pain relief and AEs, steroids were found to be the best treatment, followed by HA. Compared with placebo, single PRP, multiple PRP, and AD-MSC interventions did not result in a relevant reduction in joint pain or improvement in joint function (20). Another meta-analysis of 13 RCTs with clinical evidence level 1 also yielded similar results, showing that intra-articular MSC injection was not superior to placebo in terms of pain relief and functional improvement for patients with symptomatic knee OA (21). The reason for this discrepancy may be the substantial differences in literature search strategies among different authors, misconceptions about meta-analyses themselves, and misconceptions about the comparability of different types of stem cells in terms of their safety and regenerative potential (47).

As pluripotent stem cells, MSCs exist mainly in BM, AD and UC. All included studies examined MSCs from a single source: 8 studies examined BM-MSCs, 7 studies examined AD-MSCs, and 3 studies examined UC-MSCs. The results of the subgroup analysis showed that AD-MSCs may have a better therapeutic effect, but UC-MSCs and BM-MSCs did not have significantly stronger effects than the placebo. A network meta-analysis published in 2022 compared the effects of different sources of MSCs in the treatment of OA. Similarly, they found that compared with BM-MSCs, AD-MSCs and UC-MSCs exhibited better antiarthritic effects (48). Zhou et al. applied single-cell sequencing technology and reported that the population of AD-MSCs exhibited lower transcriptomic heterogeneity than did that of BMSCs and was less dependent on mitochondrial respiration for energy production. Furthermore, ADSCs exhibit reduced human leukocyte antigen class I antigen expression and a greater immunosuppressive capacity than BMSCs (49). Generally, AD-MSCs may be easier to obtain than the other two types of MSCs. Therefore, AD-MSCs may represent a better choice for the treatment of OA.

In the included studies, the average MSC count per injection ranged from 1× 10^6^ to 150 × 10^6^. Four studies compared different doses of MSCs with placebo in OA (14, 16, 36, 39). Gupta et al. compared the effects of 4 different doses (25, 50, 75 or 150× 10^6^ cells) of allogeneic BM-MSCs and placebo (16). They found a trend towards a reduction in pain at the lowest cell dose of 25× 10^6^ as observed by the VAS, WOMAC and ICOAP pain scoring criteria; however, these reductions were not significantly different from the effects of the placebo. Another study drew similar conclusions. When comparing 3 different doses of AD-MSCs (2, 10 or 50×10^6^ cells) for OA treatment, it was found that patients treated with 2× 10^6^ AD-MSCs experienced significant improvements in pain levels and function compared with baseline (50). Moreover, MATAS et al. conducted a comparative analysis on the effects of 3 different doses (2, 20 or 80×10^6^ cells) of UC-MSCs on OA. All three concentrations improved OA symptoms, with low and medium concentrations demonstrating greater efficacy. Additionally, 100% of the high-concentration group experienced injection-related swelling (51). These findings align closely with our own findings. Our study revealed no significant advantage of high-dose MSCs (greater than 50×10^6^ cells) compared with placebo in terms of the 6-month VAS score or 12-month VAS score.

No serious treatment-related AEs were reported in the included studies, and the most commonly reported side effects were arthralgia and swelling. We performed a meta-analysis on these 2 AEs, and the results showed that MSC injection did not increase the risk of arthralgia or swelling compared with placebo. A meta-analysis of 62 studies evaluated the safety of MSCs in different diseases (approximately 20 types of diseases). The results showed that intravenous or local implantation of MSCs increased the risk of transient fever, administration site AEs, constipation, fatigue and sleeplessness but did not increase the risk of serious AEs (52). The most notable AE was fever, which may be caused by the immunomodulatory effects of MSCs.

In general, the current research results show that MSCs have advantages in the analgesia and functional improvement of OA. The research results included in this study are very heterogeneous; thus, we conducted a sensitivity analysis and found that the outcomes and heterogeneity of the results were relatively stable after excluding one study at a time. Excessive heterogeneity may have had some effect on the authenticity of the results. We believe that the main reason for the occurrence of heterogeneity is that there are certain differences in the extraction methods of MSCs, the inclusion criteria of participants, and the selection and measurement of outcome indicators. This meta-analysis has several limitations. First, most of the follow-up periods of the included studies were relatively short, which created some difficulties in assessing the long-term efficacy of MSCs in treating OA. Second, the number of samples included in the study was generally small. Third, the number of included articles was relatively small, and the heterogeneity among the studies was high. The quality of the included studies was also poor, which may have caused a certain degree of bias. Finally, the current study was not preregistered, which may have led to selection bias, but our analysis strictly followed the systematic review process, which can reduce the risk of bias. These limitations may have a certain effect on the results, and more standardized studies are needed to solve these problems.

## 5 Conclusion

This systematic review and meta-analysis included 18 RCTs involving 1,174 participants. The present study offers preliminary evidence that local administration of MSCs derived from AD, particularly at a low dosage, can effectively alleviate pain and enhance functional outcomes in patients suffering from OA. These findings reveal the possibility of developing MSCs as drugs for the clinical treatment of OA. However, more clinical studies and more standardized experimental protocols are needed before MSCs can be applied in the clinic.

## Data availability statement

The original contributions presented in the study are included in the article/**Supplementary Material**. Further inquiries can be directed to the corresponding author.

## Author contributions

XT: Data curation, Formal analysis, Writing – original draft. ZQ: Data curation, Methodology, Software, Writing – original draft. YC: Investigation, Software, Writing – original draft. BZ: Conceptualization, Methodology, Writing – original draft.
