# Supplementary material for: Relative efficacy and safety of mesenchymal stem cells for osteoarthritis: a systematic review and meta-analysis of randomized controlled trials
Source: Front Endocrinol (Lausanne). 2024 Jun 10;15:1366297. doi: 10.3389/fendo.2024.1366297 (PMC11194387; doi:10.3389/fendo.2024.1366297)
Supplement: Supplementary file 1 [file DataSheet_1.docx]

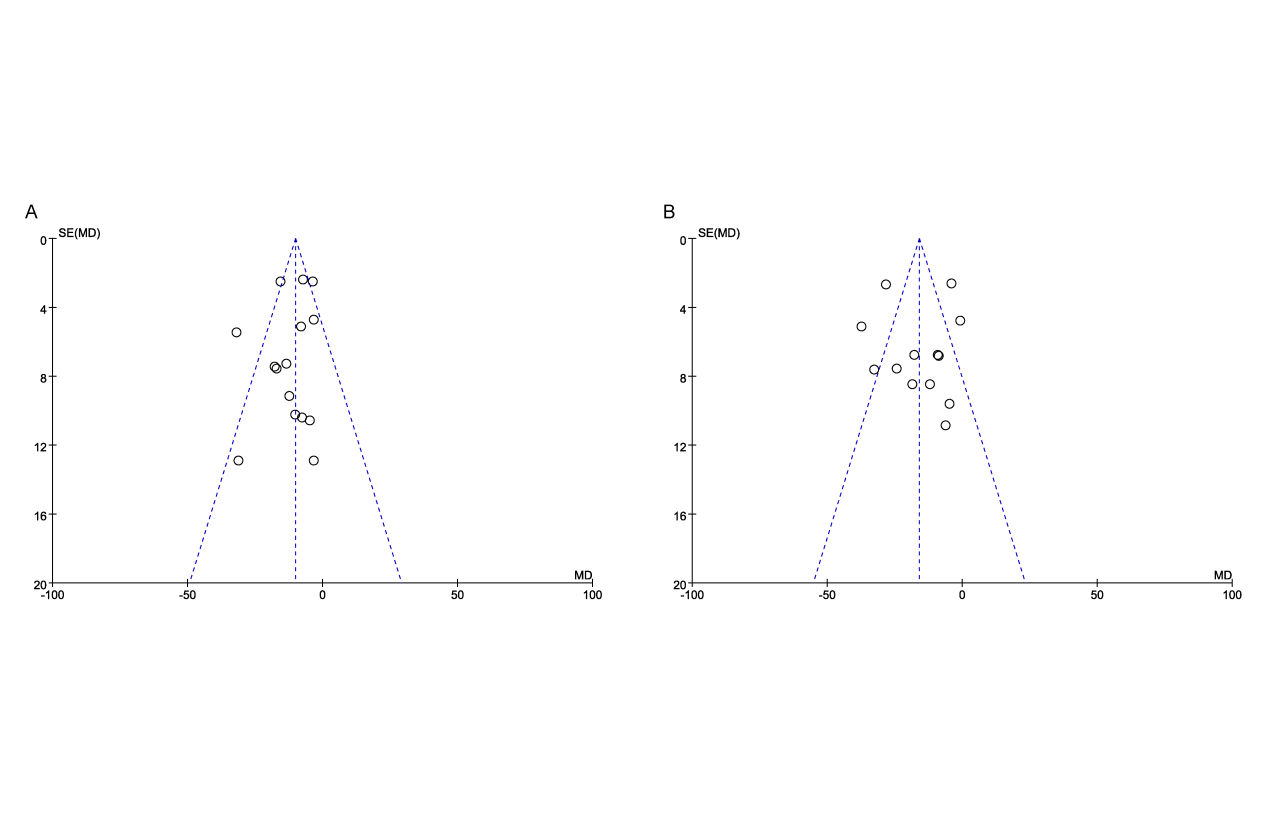


Supplementary Figure 1

Funnel plots of Western Ontario and McMaster Universities Osteoarthritis Index at 6 month(A), at 12 month(B). MD mean difference; SE standard error.


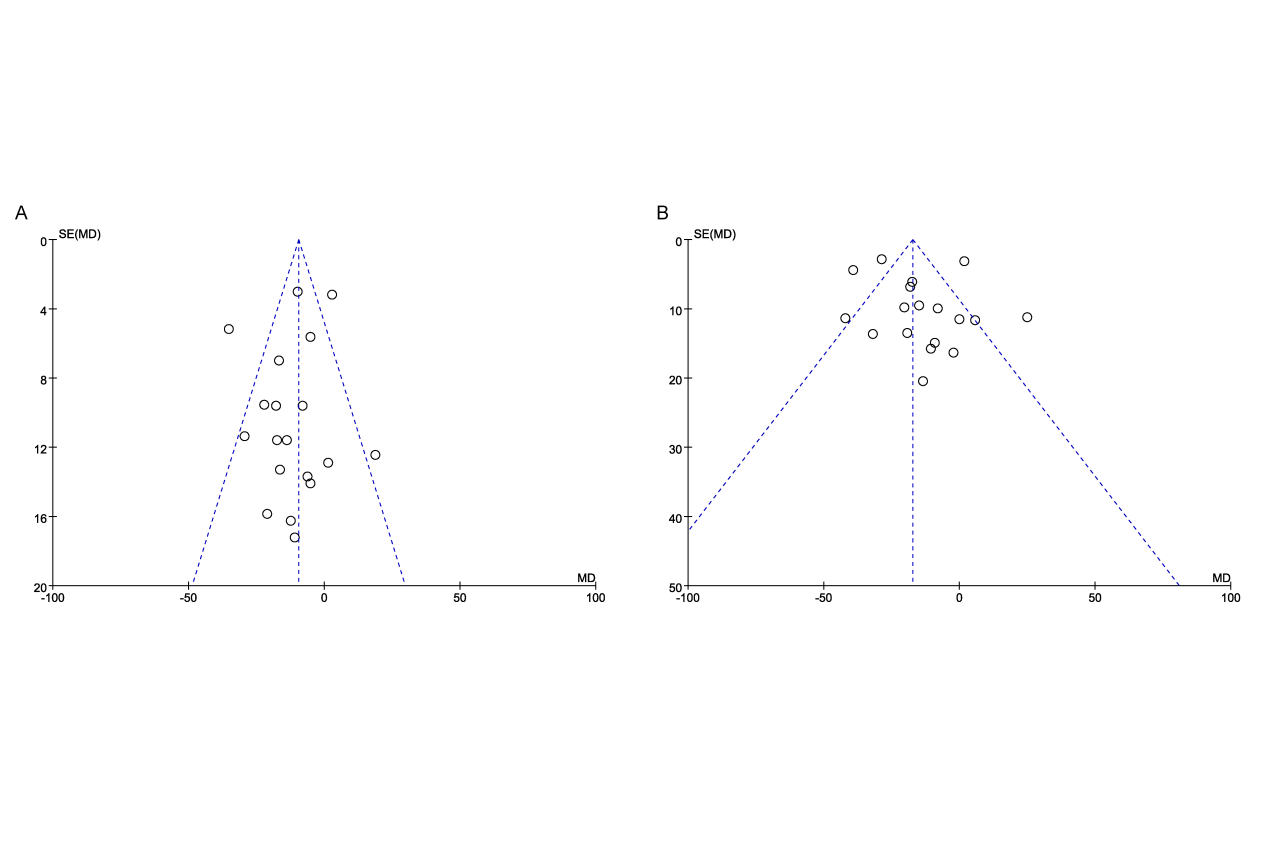


Supplementary Figure 2

Funnel plots of Visual Analog Scale at 6 month(A), at 12 month(B). MD mean difference; SE standard error.


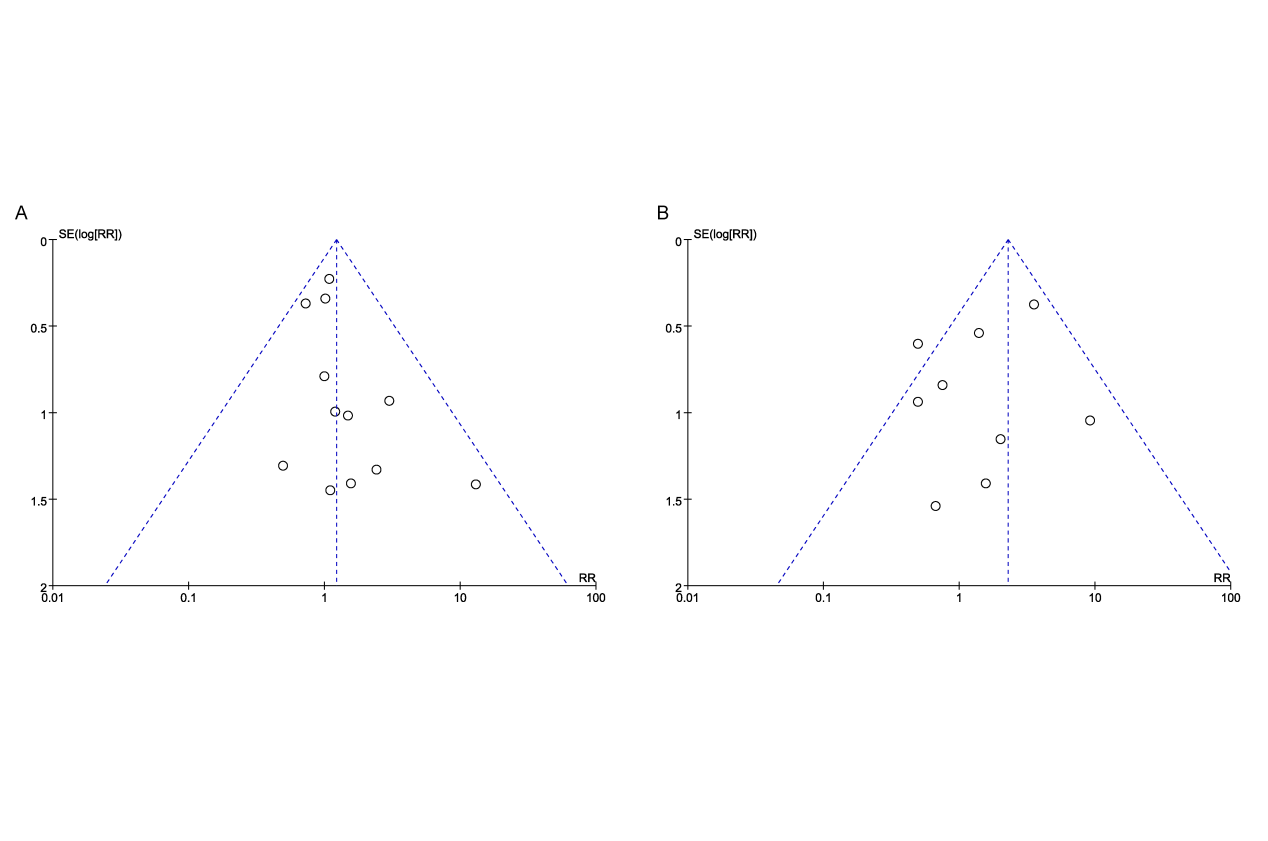


Supplementary Figure 3

Funnel plots of treatment-related adverse events, arthralgia (A), swelling (B). RR risk ratio; SE standard error.
