# Supplementary material for: Relative efficacy and safety of mesenchymal stem cells for osteoarthritis: a systematic review and meta-analysis of randomized controlled trials
Source: Front Endocrinol (Lausanne). 2024 Jun 10;15:1366297. doi: 10.3389/fendo.2024.1366297 (PMC11194387; doi:10.3389/fendo.2024.1366297)
Supplement: Supplementary file 2 [file Table_1.docx]

**S1 Table. Search strategies.**

**Source:** **Pubmed**

**Starting date:** 1946

**Searched on:** May 6, 2024

**Results:** 219

| **Search** | **Query** | **Results** |
| --- | --- | --- |
| #1 | randomized controlled trial[Publication Type] OR randomized[Title/Abstract] OR placebo[Title/Abstract] | 1,060,260 |
| #2 | "Mesenchymal Stem Cells"[Mesh] | 52,647 |
| #3 | ((((((((((((((((((((((((((((((((((((((((Mesenchymal Stem Cells[Title/Abstract]) OR (Stem Cell, Mesenchymal[Title/Abstract])) OR (Stem Cells, Mesenchymal[Title/Abstract])) OR (Mesenchymal Stem Cell[Title/Abstract])) OR (Bone Marrow Mesenchymal Stem Cells[Title/Abstract])) OR (Bone Marrow Mesenchymal Stem Cell[Title/Abstract])) OR (Bone Marrow Stromal Cells[Title/Abstract])) OR (Bone Marrow Stromal Cell[Title/Abstract])) OR (Bone Marrow Stromal Cells, Multipotent[Title/Abstract])) OR (Multipotent Bone Marrow Stromal Cell[Title/Abstract])) OR (Multipotent Bone Marrow Stromal Cells[Title/Abstract])) OR (Adipose-Derived Mesenchymal Stem Cells[Title/Abstract])) OR (Adipose Derived Mesenchymal Stem Cells[Title/Abstract])) OR (Adipose Tissue-Derived Mesenchymal Stem Cell[Title/Abstract])) OR (Adipose Tissue Derived Mesenchymal Stem Cell[Title/Abstract])) OR (Adipose Tissue-Derived Mesenchymal Stem Cells[Title/Abstract])) OR (Adipose Tissue Derived Mesenchymal Stem Cells[Title/Abstract])) OR (Mesenchymal Stem Cells, Adipose-Derived[Title/Abstract])) OR (Mesenchymal Stem Cells, Adipose Derived[Title/Abstract])) OR (Adipose Tissue-Derived Mesenchymal Stromal Cells[Title/Abstract])) OR (Adipose Tissue Derived Mesenchymal Stromal Cells[Title/Abstract])) OR (Adipose-Derived Mesenchymal Stromal Cells[Title/Abstract])) OR (Adipose Derived Mesenchymal Stromal Cells[Title/Abstract])) OR (Adipose-Derived Mesenchymal Stem Cell[Title/Abstract])) OR (Adipose Derived Mesenchymal Stem Cell[Title/Abstract])) OR (Mesenchymal Stromal Cells[Title/Abstract])) OR (Stromal Cell, Mesenchymal[Title/Abstract])) OR (Stromal Cells, Mesenchymal[Title/Abstract])) OR (Mesenchymal Stromal Cell[Title/Abstract])) OR (Multipotent Mesenchymal Stromal Cells[Title/Abstract])) OR (Mesenchymal Stromal Cells, Multipotent[Title/Abstract])) OR (Multipotent Mesenchymal Stromal Cell[Title/Abstract])) OR (Mesenchymal Progenitor Cell[Title/Abstract])) OR (Mesenchymal Progenitor Cells[Title/Abstract])) OR (Progenitor Cell, Mesenchymal[Title/Abstract])) OR (Progenitor Cells, Mesenchymal[Title/Abstract])) OR (Wharton Jelly Cells[Title/Abstract])) OR (Wharton's Jelly Cells[Title/Abstract])) OR (Wharton's Jelly Cell[Title/Abstract])) OR (Whartons Jelly Cells[Title/Abstract])) OR (Bone Marrow Stromal Stem Cells[Title/Abstract]) | 80,599 |
| #4 | #2 or #3 | 88,192 |
| #5 | "Osteoarthritis"[Mesh] | 80,521 |
| #6 | ((((((((((Osteoarthritis[Title/Abstract]) OR (Osteoarthritides[Title/Abstract])) OR (Osteoarthrosis[Title/Abstract])) OR (Osteoarthroses[Title/Abstract])) OR (Arthritis, Degenerative[Title/Abstract])) OR (Arthritides, Degenerative[Title/Abstract])) OR (Degenerative Arthritides[Title/Abstract])) OR (Degenerative Arthritis[Title/Abstract])) OR (Arthrosis[Title/Abstract])) OR (Arthroses[Title/Abstract])) OR (Osteoarthrosis Deformans[Title/Abstract]) | 105,175 |
| #7 | #5 or #6 | 125,465 |
| #8 | #1 and #4 and #7 | 219 |

**Source: EMBASE**

**Starting date:** 1974

**Searched on:** May 6, 2024

**Results:** 297

| **Search** | **Query** | **Results** |
| --- | --- | --- |
| #1 | 'osteoarthritis'/exp OR osteoarthritis:ti,ab,kw OR osteoarthritides:ti,ab,kw OR osteoosteoarthrosesarthrosis:ti,ab,kw OR 'arthritis, degenerative':ti,ab,kw OR 'arthritides, degenerative':ti,ab,kw OR 'degenerative arthritides':ti,ab,kw OR 'degenerative arthritis':ti,ab,kw OR arthrosis:ti,ab,kw OR arthroses:ti,ab,kw OR 'osteoarthrosis deformans':ti,ab,kw | 202,586 |
| #2 | 'mesenchymal stem cell'/exp OR 'mesenchymal stem cells':ti,ab,kw OR 'stem cell, mesenchymal':ti,ab,kw OR 'stem cells, mesenchymal':ti,ab,kw OR 'mesenchymal stem cell':ti,ab,kw OR 'bone marrow mesenchymal stem cells':ti,ab,kw OR 'bone marrow mesenchymal stem cell':ti,ab,kw OR 'bone marrow stromal cells':ti,ab,kw OR 'bone marrow stromal cell':ti,ab,kw OR 'bone marrow stromal cells, multipotent':ti,ab,kw OR 'multipotent bone marrow stromal cell':ti,ab,kw OR 'multipotent bone marrow stromal cells':ti,ab,kw OR 'adipose-derived mesenchymal stem cells':ti,ab,kw OR 'adipose derived mesenchymal stem cells':ti,ab,kw OR 'adipose tissue-derived mesenchymal stem cell':ti,ab,kw OR 'adipose tissue derived mesenchymal stem cell':ti,ab,kw OR 'adipose tissue-derived mesenchymal stem cells':ti,ab,kw OR 'adipose tissue derived mesenchymal stem cells':ti,ab,kw OR 'mesenchymal stem cells, adipose-derived':ti,ab,kw OR 'mesenchymal stem cells, adipose derived':ti,ab,kw OR 'adipose tissue-derived mesenchymal stromal cells':ti,ab,kw OR 'adipose tissue derived mesenchymal stromal cells':ti,ab,kw OR 'adipose-derived mesenchymal stromal cells':ti,ab,kw OR 'adipose derived mesenchymal stromal cells':ti,ab,kw OR 'adipose-derived mesenchymal stem cell':ti,ab,kw OR 'adipose derived mesenchymal stem cell':ti,ab,kw OR 'mesenchymal stromal cells':ti,ab,kw OR 'stromal cell, mesenchymal':ti,ab,kw OR 'stromal cells, mesenchymal':ti,ab,kw OR 'mesenchymal stromal cell':ti,ab,kw OR 'multipotent mesenchymal stromal cells':ti,ab,kw OR 'mesenchymal stromal cells, multipotent':ti,ab,kw OR 'multipotent mesenchymal stromal cell':ti,ab,kw OR 'mesenchymal progenitor cell':ti,ab,kw OR 'mesenchymal progenitor cells':ti,ab,kw OR 'progenitor cell, mesenchymal':ti,ab,kw OR 'progenitor cells, mesenchymal':ti,ab,kw OR 'wharton jelly cells':ti,ab,kw OR 'whartons jelly cell':ti,ab,kw OR 'whartons jelly cells':ti,ab,kw OR 'bone marrow stromal stem cells':ti,ab,kw | 127,629 |
| #3 | 'randomized controlled trial'/exp | 822,024 |
| #4 | 'randomized controlled trial':ti,ab,kw OR randomized:ti,ab,kw OR placebo:ti,ab,kw | 1,193,725 |
| #5 | #3 OR #4 | 1,469,087 |
| #6 | #1 AND #2 AND #5 | 297 |

**Source:** **Scopus**

**Starting date:** 1960

**Searched on:** May 6, 2024

**Results:** 407

| **Search** | **Query** |
| --- | --- |
| #1 | ( TITLE-ABS-KEY ( osteoarthritis ) OR TITLE-ABS-KEY ( osteoarthritides ) OR TITLE-ABS-KEY ( osteoarthrosis ) OR TITLE-ABS-KEY ( osteoarthroses ) OR TITLE-ABS-KEY ( osteoarthroses ) OR TITLE-ABS-KEY ( "Arthritides, Degenerative" ) OR TITLE-ABS-KEY ( "Degenerative Arthritides" ) OR TITLE-ABS-KEY ( "Degenerative Arthritis" ) OR TITLE-ABS-KEY ( arthrosis ) OR TITLE-ABS-KEY ( arthroses ) OR TITLE-ABS-KEY ( "Osteoarthrosis Deformans" ) ) |
| #2 | ( TITLE-ABS-KEY ( "Mesenchymal Stem Cells" ) OR TITLE-ABS-KEY ( "Stem Cell, Mesenchymal" ) OR TITLE-ABS-KEY ( "Stem Cells, Mesenchymal" ) OR TITLE-ABS-KEY ( "Mesenchymal Stem Cell" ) OR TITLE-ABS-KEY ( "Bone Marrow Mesenchymal Stem Cells" ) OR TITLE-ABS-KEY ( "Bone Marrow Mesenchymal Stem Cell" ) OR TITLE-ABS-KEY ( "Bone Marrow Stromal Cells" ) OR TITLE-ABS-KEY ( "Bone Marrow Stromal Cell" ) OR TITLE-ABS-KEY ( "Bone Marrow Stromal Cells, Multipotent" ) OR TITLE-ABS-KEY ( "Multipotent Bone Marrow Stromal Cell" ) OR TITLE-ABS-KEY ( "Multipotent Bone Marrow Stromal Cells" ) OR TITLE-ABS-KEY ( "Adipose-Derived Mesenchymal Stem Cells" ) OR TITLE-ABS-KEY ( "Adipose Derived Mesenchymal Stem Cells" ) OR TITLE-ABS-KEY ( "Adipose Tissue-Derived Mesenchymal Stem Cell" ) OR TITLE-ABS-KEY ( "Adipose Tissue Derived Mesenchymal Stem Cell" ) OR TITLE-ABS-KEY ( "Adipose Tissue-Derived Mesenchymal Stem Cells" ) OR TITLE-ABS-KEY ( "Adipose Tissue Derived Mesenchymal Stem Cells" ) OR TITLE-ABS-KEY ( "Mesenchymal Stem Cells, Adipose-Derived" ) OR TITLE-ABS-KEY ( "Mesenchymal Stem Cells, Adipose Derived" ) OR TITLE-ABS-KEY ( "Adipose Tissue-Derived Mesenchymal Stromal Cells" ) OR TITLE-ABS-KEY ( "Adipose Tissue Derived Mesenchymal Stromal Cells" ) OR TITLE-ABS-KEY ( "Adipose-Derived Mesenchymal Stromal Cells" ) OR TITLE-ABS-KEY ( "Adipose Derived Mesenchymal Stromal Cells" ) OR TITLE-ABS-KEY ( "Adipose-Derived Mesenchymal Stem Cell" ) OR TITLE-ABS-KEY ( "Adipose Derived Mesenchymal Stem Cell" ) OR TITLE-ABS-KEY ( "Mesenchymal Stromal Cells" ) OR TITLE-ABS-KEY ( "Stromal Cell, Mesenchymal" ) OR TITLE-ABS-KEY ( "Stromal Cells, Mesenchymal" ) OR TITLE-ABS-KEY ( "Mesenchymal Stromal Cell" ) OR TITLE-ABS-KEY ( "Multipotent Mesenchymal Stromal Cells" ) OR TITLE-ABS-KEY ( "Mesenchymal Stromal Cells, Multipotent" ) OR TITLE-ABS-KEY ( "Multipotent Mesenchymal Stromal Cell" ) OR TITLE-ABS-KEY ( "Mesenchymal Progenitor Cell" ) OR TITLE-ABS-KEY ( "Mesenchymal Progenitor Cells" ) OR TITLE-ABS-KEY ( "Progenitor Cell, Mesenchymal" ) OR TITLE-ABS-KEY ( "Progenitor Cells, Mesenchymal" ) OR TITLE-ABS-KEY ( "Wharton Jelly Cells" ) OR TITLE-ABS-KEY ( "Wharton's Jelly Cells" ) OR TITLE-ABS-KEY ( "Wharton's Jelly Cell" ) OR TITLE-ABS-KEY ( "Whartons Jelly Cells" ) OR TITLE-ABS-KEY ( "Bone Marrow Stromal Stem Cells" ) ) |
| #3 | ( TITLE-ABS-KEY ( randomized AND controlled AND trial ) OR TITLE-ABS-KEY ( randomized ) OR TITLE-ABS-KEY ( placebo ) ) |
| #4 | #1 AND #2 AND #3 |

**Source: Web of science**

**Starting date:** 1965

**Searched on:** May 6, 2024

**Results:** 496

| **Search** | **Query** | **Results** |
| --- | --- | --- |
| #1 | TS=(Mesenchymal Stem Cells OR Stem Cell, Mesenchymal OR Stem Cells, Mesenchymal OR Mesenchymal Stem Cell OR Bone Marrow Mesenchymal Stem Cells OR Bone Marrow Mesenchymal Stem Cell OR Bone Marrow Stromal Cells OR Bone Marrow Stromal Cell OR Bone Marrow Stromal Cells, Multipotent OR Multipotent Bone Marrow Stromal Cell OR Multipotent Bone Marrow Stromal Cells OR Adipose-Derived Mesenchymal Stem Cells OR Adipose Derived Mesenchymal Stem Cells OR Adipose Tissue-Derived Mesenchymal Stem Cell OR Adipose Tissue Derived Mesenchymal Stem Cell OR Adipose Tissue-Derived Mesenchymal Stem Cells OR Adipose Tissue Derived Mesenchymal Stem Cells OR Mesenchymal Stem Cells, Adipose-Derived OR Mesenchymal Stem Cells, Adipose Derived OR Adipose Tissue-Derived Mesenchymal Stromal Cells OR Adipose-Derived Mesenchymal Stromal Cells OR Adipose Tissue Derived Mesenchymal Stromal Cells OR Adipose Derived Mesenchymal Stromal Cells OR Adipose-Derived Mesenchymal Stem Cell OR Adipose Derived Mesenchymal Stem Cell OR Mesenchymal Stromal Cells OR Stromal Cell, Mesenchymal OR Stromal Cells, Mesenchymal OR Mesenchymal Stromal Cell OR Multipotent Mesenchymal Stromal Cells OR Mesenchymal Stromal Cells, Multipotent OR Multipotent Mesenchymal Stromal Cell OR Mesenchymal Progenitor Cells OR Mesenchymal Progenitor Cell OR Progenitor Cell, Mesenchymal OR Progenitor Cells, Mesenchymal OR Wharton Jelly Cells OR Wharton's Jelly Cells OR Wharton's Jelly Cell OR Whartons Jelly Cells OR Bone Marrow Stromal Stem Cells) | 160,092 |
| #2 | TS=(Osteoarthritis OR Osteoarthritides OR Osteoarthrosis OR Osteoarthroses OR Arthritis, Degenerative OR Arthritides, Degenerative OR Degenerative Arthritides OR Degenerative Arthritis OR Arthrosis OR Arthroses OR Osteoarthrosis Deformans) | 131,496 |
| #3 | TS=(randomized controlled trial OR controlled clinical trial OR randomized OR placebo OR randomly) | 1,727,468 |
| #4 | #1 AND #2 AND #3 | 496 |

**Source:** **Cochrane Central Register of Controlled Trials**

**Starting date:** 1998

**Searched on:** May 6, 2024

**Results:** 193

| **Search** | **Query** | **Results** |
| --- | --- | --- |
| #1 | Randomized Controlled Trial OR Double-Blind Method OR Placebos OR Random Allocation | 1,241,645 |
| #2 | MeSH descriptor: [Osteoarthritis] explode all trees | 10,913 |
| #3 | (Osteoarthritis):ti,ab,kw OR (Osteoarthritides):ti,ab,kw OR (Osteoarthrosis):ti,ab,kw OR (Osteoarthroses):ti,ab,kw OR (Arthritis, Degenerative):ti,ab,kw OR (Arthritides, Degenerative):ti,ab,kw OR (Degenerative Arthritides):ti,ab,kw OR (Degenerative Arthritis):ti,ab,kw OR (Arthrosis):ti,ab,kw OR (Arthroses):ti,ab,kw OR (Osteoarthrosis Deformans):ti,ab,kw | 24,951 |
| #4 | #2 OR #3 | 24,951 |
| #5 | MeSH descriptor: [Mesenchymal Stem Cells] explode all trees | 358 |
| #6 | (Mesenchymal Stem Cells):ti,ab,kw OR (Stem Cell, Mesenchymal):ti,ab,kw OR (Stem Cells, Mesenchymal):ti,ab,kw OR (Mesenchymal Stem Cell):ti,ab,kw OR (Bone Marrow Mesenchymal Stem Cells):ti,ab,kw OR (Bone Marrow Mesenchymal Stem Cell):ti,ab,kw OR (Bone Marrow Stromal Cells):ti,ab,kw OR (Bone Marrow Stromal Cell):ti,ab,kw OR (Bone Marrow Stromal Cells, Multipotent):ti,ab,kw OR (Multipotent Bone Marrow Stromal Cell):ti,ab,kw OR (Multipotent Bone Marrow Stromal Cells):ti,ab,kw OR (Adipose-Derived Mesenchymal Stem Cells):ti,ab,kw OR (Adipose Derived Mesenchymal Stem Cells):ti,ab,kw OR (Adipose Tissue-Derived Mesenchymal Stem Cell):ti,ab,kw OR (Adipose Tissue Derived Mesenchymal Stem Cell):ti,ab,kw OR (Adipose Tissue-Derived Mesenchymal Stem Cells):ti,ab,kw OR (Adipose Tissue Derived Mesenchymal Stem Cells):ti,ab,kw OR (Mesenchymal Stem Cells, Adipose-Derived):ti,ab,kw OR (Mesenchymal Stem Cells, Adipose Derived):ti,ab,kw OR (Adipose Tissue-Derived Mesenchymal Stromal Cells):ti,ab,kw OR (Adipose Tissue Derived Mesenchymal Stromal Cells):ti,ab,kw OR (Adipose-Derived Mesenchymal Stromal Cells):ti,ab,kw OR (Adipose Derived Mesenchymal Stromal Cells):ti,ab,kw OR (Adipose-Derived Mesenchymal Stem Cell):ti,ab,kw OR (Adipose Derived Mesenchymal Stem Cell):ti,ab,kw OR (Mesenchymal Stromal Cells):ti,ab,kw OR (Stromal Cell, Mesenchymal):ti,ab,kw OR (Stromal Cells, Mesenchymal):ti,ab,kw OR (Mesenchymal Stromal Cell):ti,ab,kw OR (Multipotent Mesenchymal Stromal Cells):ti,ab,kw OR (Mesenchymal Stromal Cells, Multipotent):ti,ab,kw OR (Multipotent Mesenchymal Stromal Cell):ti,ab,kw OR (Mesenchymal Progenitor Cell):ti,ab,kw OR (Mesenchymal Progenitor Cells):ti,ab,kw OR (Progenitor Cells, Mesenchymal):ti,ab,kw OR (Progenitor Cell, Mesenchymal):ti,ab,kw OR (Wharton Jelly Cells):ti,ab,kw OR (Wharton's Jelly Cells):ti,ab,kw OR (Wharton's Jelly Cell):ti,ab,kw OR (Whartons Jelly Cells):ti,ab,kw OR (Bone Marrow Stromal Stem Cells):ti,ab,kw | 2,521 |
| #7 | #5 OR #6 | 2,533 |
| #8 | #1 AND #4 AND #7 | 193 |
