# Supplementary material for: Relative efficacy and safety of mesenchymal stem cells for osteoarthritis: a systematic review and meta-analysis of randomized controlled trials
Source: Front Endocrinol (Lausanne). 2024 Jun 10;15:1366297. doi: 10.3389/fendo.2024.1366297 (PMC11194387; doi:10.3389/fendo.2024.1366297)
Supplement: Supplementary file 3 [file Table_2.docx]

**S2 Table. Full artical assessed for excluded.**

| Num | Reason | Intervene | Identifier | Year | Journal | Title |
| --- | --- | --- | --- | --- | --- | --- |
| 1 | incorrect study design | MFAT | 10.3390/genes10121051 | 2019 | Genes | A 24-month follow-up study of the effect of intra-articular injection of autologouss microfragmented fat tissue on proteoglycan synthesis in patients with knee osteoarthritis |
| 2 | incorrect study design | AD-MSCs | 10.5966/sctm.2015-0245 | 2016 | Stem Cells Translational Medicine | Adipose mesenchymal stromal cell-based therapy for severe osteoarthritis of the knee: A phase I dose-escalation trial |
| 3 | not relevant | AD-MSCs | 10.1016/j.arthro.2015.09.010 | 2016 | Arthroscopy | Adipose-Derived Mesenchymal Stem Cells With Microfracture Versus Microfracture Alone: 2-Year Follow-up of a Prospective Randomized Trial |
| 4 | not relevant | BM-MSCs | 10.2106/JBJS.M.00058 | 2014 | Journal of bone and joint surgery | Adult human mesenchymal stem cells delivered via intra-articular injection to the knee following partial medial meniscectomy: a randomized, double-blind, controlled study |
| 5 | not relevant | UC-MSCs | 10.1177/2325967120973052 | 2021 | Orthopaedic Journal of Sports Medicine | Allogeneic Umbilical Cord Blood–Derived Mesenchymal Stem Cell Implantation Versus Microfracture for Large, Full-Thickness Cartilage Defects in Older Patients: A Multicenter Randomized Clinical Trial and Extended 5-Year Clinical Follow-up |
| 6 | incorrect study design | UC-MSCs | 10.1007/s00264-020-04852-y | 2021 | International orthopaedics | Allogeneic umbilical cord blood-derived mesenchymal stem cells combined with high tibial osteotomy: a retrospective study on safety and early results |
| 7 | not relevant | Amniotic dehydrated cell | 10.2217/rme-2022-0005 | 2022 | Regenerative Medicine | Amniotic dehydrated cell and protein concentrate versus corticosteroid in knee osteoarthritis: preliminary findings |
| 8 | not relevant | BMAC | 10.3390/jcm8030392 | 2019 | Journal of Clinical Medicine | autologousss Matrix-Induced Chondrogenesis (AMIC) and AMIC enhanced by autologousss concentrated Bone Marrow Aspirate (BMAC) Allow for stable clinical and functional improvements at up to 9 years follow-up: Results from a Randomized controlled study |
| 9 | Combined with other treatments | Peripheral Blood Stem Cells | 10.1155/2017/8925132 | 2017 | Stem Cells International | Avoidance of Total Knee Arthroplasty in Early Osteoarthritis of the Knee with Intra-Articular Implantation of autologouss Activated Peripheral Blood Stem Cells versus Hyaluronic Acid: a Randomized Controlled Trial with Differential Effects of Growth Factor Addition |
| 10 | not relevant | BMAC | 10.1007/s00167-021-06793-4 | 2022 | KNEE | Bone marrow aspirate concentrate injections provide similar results  versus viscosupplementation up to 24 months of follow‑up in patients  with symptomatic knee osteoarthritis. A randomized controlled trial |
| 11 | not relevant | BMAC | 10.3390/medicina57111193 | 2021 | *Medicina* | Bone Marrow Aspirate Concentrate versus Platelet Rich Plasma or Hyaluronic Acid for the Treatment of Knee Osteoarthritis |
| 12 | incorrect study design | BMAC | 10.1186/s12891-021-04910-5 | 2022 | BMC Musculoskeletal Disorders | Bone marrow aspirate concentrate versus platelet-rich plasma for treating knee osteoarthritis: a one-year non-randomized retrospective comparative study |
| 13 | incorrect study design | AD-MSCs | 10.1080/03008207.2022.2074841 | 2022 | Connective Tissue Research | Clinical and laboratory findings following transplantation of allogeneic adipose-derived mesenchymal stromal cells in knee osteoarthritis, a brief report |
| 14 | not relevant | infrapatellar fat pad cell concentrates | 10.1186/s13018-021-02224-9 | 2021 | Journal of orthopaedic surgery and research | The clinical efficacy of arthroscopic therapy with knee infrapatellar fat pad cell concentrates in treating knee cartilage lesion: a prospective, randomized, and controlled study |
| 15 | not artical | SVF | 10.1177/2325967120S00127 | 2020 | Orthopaedic journal of sports medicine | Clinical efficacy of intra-articular mesenchymal stem cells for the treatment of knee osteoarthritis: a double blinded, prospective, randomized, controlled clinical trial |
| 16 | not relevant | SVF | 10.1177/0363546519899923 | 2020 | American journal of sports medicine | Clinical Efficacy of Intra-articular Mesenchymal Stromal Cells for the Treatment of Knee Osteoarthritis: a Double-Blinded Prospective Randomized Controlled Clinical Trial |
| 17 | incorrect study design | MFAT | 10.1007/s00264-020-04835-z | 2021 | International Orthopaedics | Clinical evaluation of micro-fragmented adipose tissue as a treatment option for patients with meniscus tears with osteoarthritis: a prospective pilot study |
| 18 | not relevant | SVF | 10.3389/fcell.2023.1106279 | 2023 | Frontiers in Cell and Developmental Biology | Clinical phase I/II trial of SVF therapy for cartilage regeneration: A cellular therapy with novel 3D MRI imaging for evaluating chondral defect of knee osteoarthritis |
| 19 | not relevant | SVF | 10.5966/sctm.2016-0023 | 2017 | Stem Cells Translational Medicine | Comparative Clinical Observation of Arthroscopic Microfracture in the Presence and Absence of a Stromal Vascular Fraction Injection for Osteoarthritis |
| 20 | not relevant | SVF | 10.1016/j.arthro.2014.05.036 | 2014 | Arthroscopy | Comparative outcomes of open-wedge high tibial osteotomy with platelet-rich plasma alone or in combination with mesenchymal stem cell treatment: a prospective study |
| 21 | Incorrect language | UC-MSCs | 10.7507/1002-1892.20160305 | 2016 | Chinese Journal of Reparative and Reconstructive Surgery | CURATIVE EFFECT OF HUMAN UMBILICAL CORD MESENCHYMAL STEM CELLS BY INTRA-ARTICULAR INJECTION FOR DEGENERATIVE KNEE OSTEOARTHRITIS |
| 22 | not relevant | BM-derived mononuclear cells | 10.1016/j.medici.2017.02.002 | 2017 | Medicina-Lithuania | The comparison of knee osteoarthritis treatment with single-dose bone marrow-derived mononuclear cells vs. hyaluronic acid injections |
| 23 | not relevant | BMAC | 10.2217/rme-2020-0020 | 2020 | Regenerative Medicine | Do knee injection portals affect clinical results of bone marrow aspirate concentrate injection in the treatment of osteoarthritis? A prospective randomized controlled study |
| 24 | incomplete study | AD-MSCs | [https://www.discoveryjournals.org/medicalscience/current_issue/v24/n103/A6.htm](https://www.discoveryjournals.org/medicalscience/current_issue/v24/n103/A6.htm) | 2020 | Medical Science | Effect of administration of mesenchymal stem cells on cartilage recovery and knee function in patients with Knee Osteoarthritis |
| 25 | not relevant | SVF | 10.1155/2022/2777568 | 2022 | Biomed Research International | The Effect of autologouss Adipose-Derived Stromal Vascular Fractions on Cartilage Regeneration Was Quantitatively Evaluated Based on the 3D-FS-SPGR Sequence: A Clinical Trial Study |
| 26 | not relevant | AD-MSCs | 10.14715/cmb/2021.67.3.19 | 2021 | Cellular and Molecular Biology | The effect of synthesized cartilage tissue from human adipose-derived mesenchymal stem cells in orthopedic spine surgery in patients with osteoarthritis |
| 27 | not relevant | SVF | none | 2018 | Journal of biological regulators and homeostatic agents | Evaluation of the use of autologouss micro-fragmented adipose tissue in the treatment of knee osteoarthritis: preliminary results of a randomized controlled trial |
| 28 | incorrect study design | AD-MSCs | 10.2217/rme-2017-0152 | 2018 | Regenerative medicine | Human adipose-derived mesenchymal stem cells for osteoarthritis: a pilot study with long-term follow-up and repeated injections |
| 29 | incorrect study design | Amniotic Suspension | 10.3390/jcm11123295 | 2022 | Journal of Clinical Medicine | Human Amniotic Suspension Allograft Improves Pain and Function in Knee Osteoarthritis: A Prospective Not Randomized Clinical Pilot Study |
| 30 | not relevant | BMAC | 10.1007/s00264-020-04571-4 | 2021 | International orthopaedics | Human bone marrow mesenchymal stem cell injection in subchondral lesions of knee osteoarthritis: a prospective randomized study versus contralateral arthroplasty at a mean fifteen year follow-up |
| 31 | Combined with other treatments | AD-MSCs | 10.2217/rme-2019-0068 | 2020 | Regenerative Medicine | Human adipose-derived mesenchymal progenitor cells plus microfracture and hyaluronic acid for cartilage repair: a Phase IIa trial |
| 32 | incorrect study design | SVF | 10.1007/s00167-019-05729-3 | 2020 | Knee surgery, sports traumatology, arthroscopy | Implantation of mesenchymal stem cells in combination with allogenic cartilage improves cartilage regeneration and clinical outcomes in patients with concomitant high tibial osteotomy |
| 33 | incorrect study design | AD-MSCs | 10.1016/j.knee.2012.04.001 | 2012 | Knee | Infrapatellar fat pad-derived mesenchymal stem cell therapy for knee osteoarthritis |
| 34 | Combined with other treatments | BM-MSCs | 10.1016/j.arthro.2013.09.074 | 2013 | Arthroscopy | Injectable cultured bone marrow-derived mesenchymal stem cells in varus knees with cartilage defects undergoing high tibial osteotomy: A prospective, randomized controlled clinical trial with 2 years' follow-up |
| 35 | retracted artical | SVF | 10.1186/s12967-017-1242-4 | 2017 | Journal of Translational Medicine | Intra-articular injection in the knee of adipose derived stromal cells (stromal vascular fraction) and platelet rich plasma for osteoarthritis |
| 36 | incorrect study design | AD-MSCs | 10.1002/jgm.3002 | 2018 | Journal of Gene Medicine | Intra-articular injection of autologouss adipose-derived mesenchymal stem cells in the treatment of knee osteoarthritis |
| 37 | not relevant | SVF | 10.1007/s00264-018-4099-0 | 2019 | International Orthopaedics | Intra-articular injection of autologouss adipose-derived stromal vascular fractions for knee osteoarthritis: a double-blind randomized self-controlled trial |
| 38 | Combined with other treatments | AD-MSCs | 10.1093/stcltm/szac023 | 2022 | Stem cells translational medicine | Intra-articular Injection of Mesenchymal Stem Cells after High Tibial Osteotomy in Osteoarthritic Knee: two-Year Follow-up of Randomized Control Trial |
| 39 | incorrect study design | AD-MSCs | 10.1177/0363546517716641 | 2017 | American Journal of Sports Medicine | Intra-articular Injection of Mesenchymal Stem Cells for the Treatment of Osteoarthritis of the Knee A 2-Year Follow-up Study |
| 40 | not artical | BM-MSCs | 10.1186/s12967-018-1591-7 | 2018 | Journal of translational medicine | Intra-articular injection of two different doses of autologouss bone marrow mesenchymal stem cells versus hyaluronic acid in the treatment of knee osteoarthritis: long-term follow up of a multicenter randomized controlled clinical trial (phase I/II) |
| 41 | incorrect study design | AD-MSCs | 10.2217/rme-2019-0106 | 2020 | Regenerative Medicine | Intra-articular injections of allogeneic human adipose-derived mesenchymal progenitor cells in patients with symptomatic bilateral knee osteoarthritis: a Phase I pilot study |
| 42 | not relevant | MFAT | 10.3390/biomedicines10030684 | 2022 | Biomedicines | Intra-Articular Injections of autologouss Adipose Tissue or Platelet-Rich Plasma Comparably Improve Clinical and Functional Outcomes in Patients with Knee Osteoarthritis |
| 43 | incorrect study design | BM-MSCs | 10.1007/s00167-018-4883-9 | 2018 | Knee surgery, sports traumatology, arthroscopy | Intra-articular injections of expanded mesenchymal stem cells with and without addition of platelet-rich plasma are safe and effective for knee osteoarthritis |
| 44 | not relevant | BMAC | 10.21823/2311-2905-1669 | 2021 | Travmatologiya I Ortopediya Rossii | Intraosseous Injection of autologouss Bone Marrow Aspirate Concentrate and PlateLet-Rich Plasma for Treatment of Knee Osteoarthritis |
| 45 | not relevant | synovia-MSC | 10.1007/s00402-014-2136-z | 2015 | Archives of Orthopaedic and Trauma Surgery | Matrix-induced autologouss mesenchymal stem cell implantation versus matrix-induced autologouss chondrocyte implantation in the treatment of chondral defects of the knee: a 2-year randomized study |
| 46 | not relevant | MFAT | 10.1007/s00167-022-07101-4 | 2022 | Knee Surgery Sports Traumatology Arthroscopy | Micro-fragmented adipose tissue (mFAT) associated with arthroscopic debridement provides functional improvement in knee osteoarthritis: a randomized controlled trial |
| 47 | not relevant | MFAT | 10.1177/03635465221115821 | 2022 | The American journal of sports medicine | Microfragmented Adipose Tissue Versus Platelet-Rich Plasma for the Treatment of Knee Osteoarthritis: A Prospective Randomized Controlled Trial at 2-Year Follow-up |
| 48 | not relevant | SVF | 10.1186/s13287-022-02788-1 | 2022 | Stem Cell Research & Therapy | Mid-term prognosis of the stromal vascular fraction for knee osteoarthritis: a minimum 5-year follow-up study |
| 49 | not relevant | chondrocytes | 10.1055/s-0036-1597275 | 2017 | Journal of Knee Surgery | An MRI Evaluation of Patients Who Underwent Treatment with a Cell-Mediated Gene Therapy for Degenerative Knee Arthritis: A Phase IIa Clinical Trial |
| 50 | incorrect study design | AD-MSCs | 10.1186/s13287-019-1406-7 | 2019 | Stem Cell Research & Therapy | Multi-compositional MRI evaluation of repair cartilage in knee osteoarthritis with treatment of allogeneic human adipose-derived mesenchymal progenitor cells |
| 51 | incorrect study design | BM-MSCs | none | 2012 | Annals of the Academy of Medicine Singapore | A novel, minimally-invasive technique of cartilage repair in the human knee using arthroscopic microfracture and injections of mesenchymal stem cells and hyaluronic acid-a prospective comparative study on safety and short-term efficacy |
| 52 | Combined with other treatments | BMAC | none | 2010 | [Journal of the Indian Medical Association](https://xueshu.baidu.com/usercenter/data/journal?cmd=jump&tn=SE_baiduxueshu_c1gjeupa&ie=utf-8&sc_f_para=sc_hilight=publish&sort=sc_cited&wd=journaluri:(15926a80234caa3c) Journal of the Indian Medical Association" \o "https://xueshu.baidu.com/usercenter/data/journal?cmd=jump&tn=SE_baiduxueshu_c1gjeupa&ie=utf-8&sc_f_para=sc_hilight=publish&sort=sc_cited&wd=journaluri:(15926a80234caa3c) Journal of the Indian Medical Association) | The new avenues in the management of osteo-arthritis of knee--stem cells |
| 53 | not relevant | BMAC | 10.1177/0363546516656179 | 2016 | American Journal of Sports Medicine | One-Stage Cartilage Repair Using a Hyaluronic Acid-Based Scaffold With Activated Bone Marrow-Derived Mesenchymal Stem Cells Compared With Microfracture Five-Year Follow-up |
| 54 | not relevant | SVF | 10.7759/cureus.34595 | 2023 | Cureus | Outcome of Intra-articular Injection of Total Stromal Cells and Platelet-Rich Plasma in Primary Knee Osteoarthritis: A Randomized Clinical Trial |
| 55 | not relevant | AD-MSCs | 10.1155/2022/6048477 | 2022 | Advances in orthopedics | Platelet-Rich Plasma and Adipose-Derived Mesenchymal Stem Cells in Association with Arthroscopic Microfracture of Knee Articular Cartilage Defects: a Pilot Randomized Controlled Trial |
| 56 | not relevant | MFAT | 10.1177/23259671221120678 | 2022 | Orthopaedic journal of sports medicine | Platelet-Rich Plasma Versus Microfragmented Adipose Tissue for Knee Osteoarthritis: a Randomized Controlled Trial |
| 57 | incorrect study design | BMAC | 10.1016/j.knee.2020.08.018 | 2020 | Knee | Positive early clinical outcomes of bone marrow aspirate concentrate for osteoarthritis using a novel fenestrated trocar |
| 58 | not relevant | MFAT | 10.1089/scd.2021.0053 | 2021 | Stem Cells and Development | A Prospective Study Comparing Leukocyte-Poor Platelet-Rich Plasma Combined with Hyaluronic Acid and autologouss Microfragmented Adipose Tissue in Patients with Early Knee Osteoarthritis |
| 59 | not relevant | BMAC | 10.1177/0363546516662455 | 2017 | American Journal of Sports Medicine | A Prospective, Single-Blind, Placebo-Controlled Trial of Bone Marrow Aspirate Concentrate for Knee Osteoarthritis |
| 60 | incorrect study design | BMAC | 10.1177/1947603518796142 | 2019 | Cartilage | Quantitative T2 MRI Mapping and 12-Month Follow-up in a Randomized, Blinded, Placebo Controlled Trial of Bone Marrow Aspiration and Concentration for Osteoarthritis of the Knees |
| 61 | incorrect study design | UC-MSCs | 10.1016/j.jcjp.2021.100037 | 2022 | Journal of Cartilage and Joint Preservation | Safety of an allogeneic, human, umbilical cord blood-derived mesenchymal stem cells-4% hyaluronate composite for cartilage repair in the knee |
| 62 | not relevant | BMAC | 10.46582/JSRM.1701002 | 2021 | Journal of stem cells & regenerative medicine | A single-blinded randomized controlled trial of mesenchymal stem cell therapy for the treatment of osteoarthritis of the knee with active control |
| 63 | not relevant | BMAC | 10.1186/s12967-018-1736-8 | 2018 | Journal of Translational Medicine | A specific protocol of autologouss bone marrow concentrate and platelet products versus exercise therapy for symptomatic knee osteoarthritis: a randomized controlled trial with 2year follow-up |
| 64 | not relevant | BMAC | 10.1007/s00264-020-04687-7 | 2021 | International Orthopaedics | Subchondral bone or intra-articular injection of bone marrow concentrate mesenchymal stem cells in bilateral knee osteoarthritis: what better postpone knee arthroplasty at fifteen years? A randomized study |
| 65 | not relevant | BMAC | 10.1007/s00264-018-3916-9 | 2018 | International orthopaedics | Subchondral stem cell therapy versus contralateral total knee arthroplasty for osteoarthritis following secondary osteonecrosis of the knee |
| 66 | incorrect study design | SVF | 10.3390/cells8040308 | 2019 | Cells | Time-and kellgren-lawrence grade-dependent changes in intra-articularly transplanted stromal vascular fraction in osteoarthritic patients |
| 67 | not relevant | BM-MSCs | 10.1016/j.reth.2019.06.002 | 2019 | Regenerative therapy | Transplantation of autologouss bone marrow-derived mesenchymal stem cells under arthroscopic surgery with microfracture versus microfracture alone for articular cartilage lesions in the knee: a multicenter prospective randomized control clinical trial |
| 68 | incorrect study design | MFAT | 10.1007/s00264-021-05093-3 | 2021 | International Orthopaedics | The use of intra-articular injection of autologous micro-fragmented adipose tissue as pain treatment for ankle osteoarthritis: a prospective not randomized clinical study |

AD= Adipose tissue, BM= Bone marrow, UC= Umbilical cord blood, MSC= Mesenchymal stem cell, MFAT= Micro-fragmented fat tissue, BMAC= Bone marrow aspirate concentrate, SVF= Stromal vascular fraction stem cell
